# Potential of novel *Mycobacterium tuberculosis *infection phase-dependent antigens in the diagnosis of TB disease in a high burden setting

**DOI:** 10.1186/1471-2334-12-10

**Published:** 2012-01-20

**Authors:** Novel N Chegou, Gillian F Black, Andre G Loxton, Kim Stanley, Paulin N Essone, Michel R Klein, Shreemanta K Parida, Stefan HE Kaufmann, T Mark Doherty, Annemieke H Friggen, Kees L Franken, Tom H Ottenhoff, Gerhard Walzl

**Affiliations:** 1 DST/NRF Centre of Excellence for Biomedical Tuberculosis Research and MRC Centre for Molecular and Cellular Biology, Division of Molecular Biology and Human Genetics, Department of Biomedical Sciences, Faculty of Health Sciences, Stellenbosch University, P.O. Box 19063, Tygerberg 7505, South Africa; 2 Department of Infectious Diseases, Leiden University Medical Centre, P.O. Box 9600, 2300 RC Leiden, The Netherlands; 3 Department of Immunology, Max Planck Institute for Infection Biology, Charitéplatz 1, 10117 Berlin, Germany; 4 Department of Infectious Disease Immunology, Statens Serum Institute, Copenhagen 2300s, Denmark; 5 GlaxoSmithKline, Nykær 68, Copenhagen, Denmark

## Abstract

**Background:**

Confirming tuberculosis (TB) disease in suspects in resource limited settings is challenging and calls for the development of more suitable diagnostic tools. Different *Mycobacterium tuberculosis (M.tb) *infection phase-dependent antigens may be differentially recognized in infected and diseased individuals and therefore useful as diagnostic tools for differentiating between *M.tb *infection states. In this study, we assessed the diagnostic potential of 118 different *M.tb *infection phase-dependent antigens in TB patients and household contacts (HHCs) in a high-burden setting.

**Methods:**

Antigens were evaluated using the 7-day whole blood culture technique in 23 pulmonary TB patients and in 19 to 21 HHCs (total n = 101), who were recruited from a high-TB incidence community in Cape Town, South Africa. Interferon-gamma (IFN-γ) levels in culture supernatants were determined by ELISA.

**Results:**

Eight classical TB vaccine candidate antigens, 51 DosR regulon encoded antigens, 23 TB reactivation antigens, 5 TB resuscitation promoting factors (rpfs), 6 starvation and 24 other stress response-associated TB antigens were evaluated in the study. The most promising antigens for ascertaining active TB were the rpfs (Rv0867c, Rv2389c, Rv2450c, Rv1009 and Rv1884c), with Areas under the receiver operating characteristics curves (AUCs) between 0.72 and 0.80. A combination of *M.tb *specific ESAT-6/CFP-10 fusion protein, Rv2624c and Rv0867c accurately predicted 73% of the TB patients and 80% of the non-TB cases after cross validation.

**Conclusions:**

IFN-γ responses to TB rpfs show promise as TB diagnostic candidates and should be evaluated further for discrimination between *M.tb *infection states.

## Background

The diagnosis of tuberculosis (TB) disease remains a challenge in resource-limited settings. This is particularly due to limitations in sputum smear microscopy [1], the cheapest and widely used test in such settings. Several attempts have been made towards the development of better diagnostic tests for TB [1]. The most significant development in the field has been the introduction of the automated real-time sputum processing molecular beacon assay, XpertMTB/RIF assay (Cepheid Inc., CA, USA) [2] into clinical practice but the relatively high operating costs preclude its use in resource-poor settings which often suffer from the highest TB burdens [3]. Furthermore, the dependence of most of these tests on sputum implies that they are not suitable for patients with difficulties in providing good quality sputum samples such as children, extrapulmonary TB cases, or in cases where the sputum itself is negative (for example, many patients with HIV-*Mycobacterium tuberculosis (M.tb) *co-infections). Immunodiagnostic assays might hold promise in such cases [4] especially if they can be developed into point-of-care tests.

One of the most notable advances in the development of immunodiagnostic tools for *M.tb *has been the advent of the interferon gamma release assays (IGRAs). Commercially available IGRAs include the ELISA-based Quantiferon TB Gold assays (Cellestis, Victoria, Australia) and the ELISPOT-based T SPOT.TB test (Oxford Immunotec, Abington, UK). Notwithstanding the proven usefulness of these assays in the diagnosis of *M.tb *infection, especially in comparison to the tuberculin skin test (TST) [5-7], an important limitation of the tests is that they do not discriminate between latent *M.tb *infection (LTBI) and active TB disease. This implies limited utility in high-burden settings where the prevalence of LTBI is usually high. In order to develop T cell-based assays for the diagnosis of active TB, it is imperative to identify new host markers expressed in response to established *M.tb*-dependent antigens, such as those used in IGRAs (ESAT-6, CFP-10, TB7.7), or novel *M.tb *antigens uniquely recognized by patients with active TB or LTBI.

In response to the continued pressure mounted by host immune responses in the course of *M.tb *infection, the bacterium is believed to respond by self-regulating the expression of many genes [8,9]. Using *in vivo *and *in vitro *models believed to mimic the conditions encountered within the host by the tubercle bacillus as infection progresses from latency to active disease, investigators have been able to identify infection phase-dependent genes with diagnostic potential [10,11]. In the present study, we assessed the potential diagnostic utilities of 118 *M.tb *infection phase-dependent antigens in TB patients and household contacts (HHCs) in a high-TB burden population. As part of a large ongoing TB biomarker study, we investigated the antigens in a 7-day diluted whole blood assay for their diagnostic potential for active TB.

## Methods

### Study subjects

Participants were recruited from the Ravensmead/Uitsig community in Cape Town, South Africa, as part of an ongoing Bill and Melinda Gates Foundation funded Grand Challenges in Global Health (GC6-74) study (http://www.biomarkers-for-tb.net/). Participants included in this study were enrolled between October 2006 and April 2007. All TB patients were self-reporting, untreated cases with a first episode of TB and were acid fast bacilli (AFB) positive on two sputum smears. HHCs had been living in the same house as an adult TB case who was diagnosed not more than 2 months before recruitment of the contact. All HHCs had antero-posterior view chest X-rays and assisted sputum samples taken. The X-rays were all evaluated as normal and the sputum samples found negative for AFB. Demographic data was collected on all participants and a clinical questionnaire completed. All participants tested negative with an HIV rapid test (Abbot Determine™ HIV 1/2; Abbott, Wiesbaden, Germany). Inclusion criteria for all participants were: age between 18 and 60 years, willingness to undergo HIV testing and to give written informed consent for participation in the study. Exclusion criteria for all participants included HIV infection, previous or current TB treatment, participation in a drug or vaccine trial at or within the 6 months preceding recruitment, other chronic illnesses and pregnancy. At enrolment, 10 ml of heparinized blood was collected from all participants and transported within 2 h of collection to the laboratory where a 7-day whole blood assay (WBA) (described below) was performed. Ethical approval for the study was obtained from the Committee for Human Research of the University of Stellenbosch.

### Antigens

A total of 118 *M.tb *infection phase-dependent antigens (112 recombinant proteins and 8 peptide pools) were evaluated in the study. Eight of these antigens (ESAT-6/CFP-10 fusion protein (ESAT-6/CFP-10fp), TB10.4 (Rv0288), TB10.3 (Rv3019c), TB7.7 (Rv2654c), Ag85A (Rv3804c), Ag85B (Rv1886c), HSP65 (Rv0440), ESAT-6(Rv3875)), were well-characterized TB antigens and were evaluated as "control antigens". Fifty-one antigens were DosR-induced antigens, 23 were reactivation antigens, 5 were rpfs, 6 were starvation antigens (other than Rv2654c) and the other 24 are known to be associated with stress conditions (Additional file 1: Tables S1, Additional file 2: Table S2, Additional file 3: Table S3, Additional file 4: Table S4, Additional file 5: Table S5, Additional file 6: Table S6). One of the peptide pools (Rv2659) was evaluated in three separate pools (pools A-C) while Ag85A and Ag85B were evaluated together in culture. With the exception of ESAT-6 and Rv3407, all the recombinant proteins were produced at Leiden University Medical Center (LUMC), The Netherlands. Recombinant ESAT-6 and all peptide pools were from SSI, and Rv3407 from the Max Plank Institute for Infection Biology (Berlin, Germany).

For the antigens that were available as peptide pools, there were 6 to 13 peptides in each pool. Each of the peptides was 15 amino acids long. There were eight amino acid overlaps between the peptides in each pool (Additional file 7: Table S7).

Recombinant antigens were produced as previously described [12]. Briefly, *M.tb *genes were amplified by PCR from genomic DNA of *M.tb *and cloned using the Gateway technology platform (Invitrogen, Carlsbad, CA) with pDEST17 expression vector containing an N-terminal histidine tag (Invitrogen). Sequencing was performed on selected clones to confirm identity of all cloned DNA fragments. Recombinant proteins were over expressed in *E. coli *BL21(DE3) and purified as described to remove any traces of endotoxin. Each purified recombinant protein was analyzed by 12% SDS-PAGE followed by Coomassie Brilliant Blue staining and Western-blotting with an anti-His antibody (Invitrogen) to confirm size and purity. Endotoxin contents were below 50 IU/mg recombinant protein as tested using a Limulus Amebocyte Lysate (LAL) assay (Cambrex, East Rutherford, NJ), and less than 0.1 IU/mg in some cases. Recombinant proteins were tested to exclude protein non-specific T cell stimulation and cellular toxicity in IFN-γ release assays using PBMC of *in vitro *PPD-negative, healthy Dutch donors recruited at the Blood bank Sanquin, Leiden, The Netherlands. None of these controls had experienced any known prior contact with TB patients.

### Whole blood assay

Antigens were evaluated at a final concentration of 10 μg/ml as previously described [13]. Briefly, antigens were reconstituted as instructed by the respective manufacturers, to a concentration of 20 μg/ml, after which they were aliquoted into 96-well U-bottom plates (100 μl per well) in triplicates. Antigen plates were then frozen at -80°C until the day of WBA. On the day of WBA, antigen plates were thawed and 100 μl of 1 in 5 diluted whole blood [diluted with pre-warmed (37°C) medium (RPMI 1640 containing L-glutamine) (Sigma)], was added to the antigen and control wells. Plates were then incubated until day 7 in a humidified, 5% CO_2_, 37°C incubator. 150 μl of supernatant was harvested from each of the three antigen/control wells, pooled in a micro centrifuge tube (total of 450 μl per antigen per study participant), divided into 3 aliquots and frozen at -80°C until further use. The unstimulated control wells received medium without antigen while the positive control wells received phytohaemagglutinin (PHA) (Sigma) at 0.5 μg/ml.

### IFN-γ ELISA

ELISAs were performed using a pair of monoclonal antibodies from BD Pharmingen™, (BD Biosciences). Briefly, 96-well flat-bottomed micro-titre plates (Nunc) were coated with 2 μg/ml (50 μl) of the purified mouse anti-human IFN-γ monoclonal capture antibody (diluted in 0.1 M NaHCO_3_). After overnight incubation at 4°C, plates were washed and blocked with 10% heat-inactivated fetal calf serum (150 μl) in phosphate buffered saline for 2 h (room temperature). After another wash, serial dilutions of recombinant human IFN-γ standard (BD Pharmingen™), and the samples (100 μl per well), were added to the designated wells, and plates incubated overnight at 4°C. Plates were then washed and 1 μg/ml (100 μl) of the biotinylated mouse-anti-human IFN-γ detection antibody (BD Pharmingen™) added. After 1 h incubation (room temperature) and a further wash, 2.5 μg/ml (100 μl) of streptavidin peroxidase (Sigma) was added to all wells, followed by addition of the substrate (O-phenylene diamine-dihydrochloride [Sigma]) (200 μl/well). Plates were then incubated in the dark for 25 mins and the optical densities read at 450 nm. All samples, standards and controls were assayed in duplicate. IFN-γ concentrations were calculated from a four-parameter logistic curve using the microplate manager software (Bio Rad Laboratories). A PHA-stimulated internal positive control supernatant (evaluated on all plates to assess the variability of the ELISA assay) had an average interplate coefficient of variation of 3.6% (95% confidence interval, 2.7-4.6%).

Although the ELISA used in our study has been used in other published studies [13-15], the standard curve for the assay ranged from 31-4000 pg/ml and many of the responses elicited by the antigens we have tested fell below the lowest point on the curve. To verify our observations, we re-evaluated the supernatants from promising antigens by a commercially available IFN-γ ELISA kit (Quantiferon TB Gold (QFT) ELISA, Cellestis, Australia). According to the manufacturer, the detection limit of the QFT ELISA is 0.05 IU/ml (about 2 pg/ml). The validation ELISA (QFT ELISA) was performed according to the manufacturer's instructions.

### Statistical analysis

IFN-γ responses obtained with the Quantiferon ELISA (in IU/ml) were converted to pg/ml by multiplying by 40 [16]. Differences in IFN-γ levels elicited by the antigens were evaluated by the Mann Whitney U test for nonparametric data analysis. Optimal cut-off levels for differentiating between TB disease and no TB were ascertained by receiver operating characteristics (ROC) analysis using the "R" statistical programming language. The predictive abilities of combinations of antigens to differentiate between TB disease and no TB was investigated by performing best subsets general discriminant analysis (GDA), with leave-one-out cross validation as described previously [17]. A 5% significance level was used as guideline for determining significant associations. The data were analyzed using the Statistica 8 software, (Statsoft, Ohio, USA) and GraphPad prism, version 5.00 for Windows (GraphPad Software, San Diego, California, USA).

## Results

A total of 124 participants, 23 TB patients and 101 HHCs were enrolled in the study. The mean (± standard deviation) age of participants was 31.7 (± 15.3) years and 76 (61.3%) were female. Although neither IGRA nor TST results were available for the HHCs, it is known from our previous studies that 78-89% of adult HIV-negative TB HHCs from the study community are TST positive (10 mm cut-off) with a mean TST induration of about 23 mm [5,13,17]. All the antigens (n = 118) were evaluated in all TB patients (n = 23) and in groups of 19 to 21 HHCs. Because of the large number of antigens evaluated in the study, only antigens for which reportable data was generated are shown in the results section of the main manuscript. The diagnostic accuracy data for all the other antigens is shown in Additional file 8: Table S8 and Additional file 9: Figure S1. The detailed description of all the antigens evaluated in the study is shown in Additional file 1: Table S1, Additional file 2: Table S2, Additional file 3: Table S3, Additional file 4: Table S4, Additional file 5: Table S5, Additional file 6: Table S6, Additional file 7: Table S7.

### IFN-γ responses in assay controls

The median IFN-γ concentration detected in the unstimulated control supernatants in both TB patients and HHCs was 0 pg/ml (range 0.0-4.3 pg/ml in TB patients and 0.0-585.4 pg/ml in HHCs). There was no significant difference in the unstimulated IFN-γ responses between the TB patients and HHCs (*p *= 0.10). The median IFN-γ concentration detected in PHA-stimulated supernatants in HHCs (1331.0 pg/ml, range, 0.0 to > 4000 pg/ml), was significantly higher than that observed in TB patients (326.6 pg/ml, range, 0.0 to > 4000 pg/ml) (*p *= 0.01), as previously observed [18]. The IFN-γ response obtained for the unstimulated control for each participant was subtracted from all antigen/control-stimulated levels before further analysis of the data.

### IFN-γ production in response to classical TB antigens

Eight *M.tb *classical antigens (Additional file 1: Table S1) were evaluated in this study. TB10.4 and TB7.7 were available as peptide pools while all other classical antigens were recombinant proteins. Ag85A and Ag85B were pooled and tested as a single condition.

**Figure 1:**
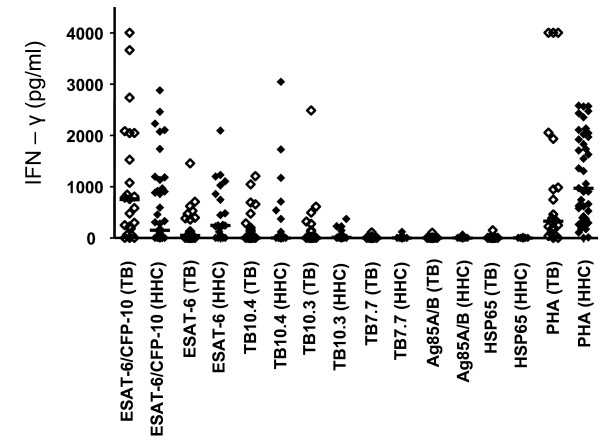
**IFN-γ levels (pg/ml) elicited by classical TB antigens in 23 TB patients (TB) and 20 household contacts (HHC)**. Responses in TB cases are indicated by open symbols and those in household contacts by closed symbols. Error bars represent the median. Ag85A/B = Ag85A and Ag85B tested together, HSP = heat shock protein, PHA = Phytohaemagglutinin.

ESAT-6/CFP-10fp, ESAT-6, TB10.4 and TB10.3 were the most frequently recognized antigens in TB patients and HHCs. Recognition of TB7.7, Ag85A and B and HSP65 was poor in both subject groups (Figure 1). There were no significant differences in the IFN-γ responses elicited by any of the antigens between the two study groups (*p *values all > 0.05, range, 0.06-0.98). Similarly, when the ability of the antigens to differentiate between TB patients and HHCs was evaluated using ROC analysis, the antigen with the highest area under the ROC curve (AUC) was ESAT-6 (AUC of 0.66). The AUCs for all the classical TB antigens were between 0.44 and 0.66 (Additional file 8: Table S8, Additional file 9: Figure S1).

### DosR regulon encoded antigens

Fifty-one DosR regulon encoded antigens (Additional file 2: Table S2) were evaluated in this study. All antigens were recombinant proteins. These antigens were more frequently recognized in HHCs, confirming previous work [13,19-21]. When antigen induced IFN-γ levels were compared between the two subject groups, the difference in IFN-γ responses were significant for 14 of the 51 antigens evaluated, namely: Rv1735c, Rv2006, Rv2625c, Rv1996, Rv2032, Rv2629, Rv3126c, Rv0081, Rv2631, Rv3130c, Rv2624c, Rv2007c, Rv2028c and Rv3134 (Table 1). The p-values for the differences in IFN-γ for six other antigens showed a trend for differential IFN-γ induction (range of p-values, 0.06 - 0.08), namely: Rv2029c, Rv2030c, Rv3129, Rv2630, Rv3133c and Rv3127 (Additional file 8: Table S8).

**Table 1:** Abilities of DosR antigens to discriminate between TB disease and no TB.

Antigen	TB cases	HHCs	P-value	AUC	Cut-off value	Sensitivity (%)	Specificity (%)
Rv1735c	0.0 (0.0-140.9	12.0 (0.0-1049.0)	0.040	0.68	< 6	83 (19/23)	52 (11/21)

**Rv2625c**	**0.0(0.0-1784.0)**	**17.3(0.0-1121.0)**	**0.007**	**0.74**	**< 6.1**	**83 (19/23)**	**70 (14/20)**

**Rv1996**	**0.0(0.0-180.6)**	**5.0(0.0-** **314.1)**	**0.004**	**0.75**	**< 0.5**	**83 (19/23)**	**76 (16/21)**

**Rv2032**	**0.0(0.0-39.2)**	**17.7(0.0-** **331.6)**	**0.002**	**0.77**	**< 0.1**	**83 (19/23)**	**70 (14/20)**

Rv3126c	0.0(0.0-1173.0)	4.6(0.0- 820.6)	0.030	0.69	< 4.6	87 (20/23)	50 (10/20)

**Rv0081**	**0.0(0.0-53.6)**	**5.5(0.0-** **732.1)**	**0.020**	**0.71**	**< 9.4**	**96 (22/23)**	**50 (10/20)**

Rv3130c	0.0(0.0-41.9)	0.0(0.0- 67.4)	0.050	68.0	< 1.1	91 (21/23)	45 (9/20)

**Rv2624c**	**0.0(0.0-288.6)**	**3.4(0.0-** **78.7)**	**0.010**	**0.73**	**< 1.0**	**91 (21/23)**	**55 (11/20)**

Rv2028c	0.0(0.0-174.9)	0.0(0.0- 72.5)	0.030	0.69	< 2.3	96 (22/23)	45 (9/20)

Rv3134c	0.0(0.0-0.0)	0.0(0.0- 51.2)	0.050	0.68	< 4.9	100 (23/23)	35 (7/20)

**Rv2006**	**0.0 (0.0-2738.0)**	**12.3 (0.0-1030.0)**	**0.002**	**0.77**	**< 1.0**	**83 (19/23)**	**80 (16/20)**

**Rv2629**	**0.0 (0.0-1720.0)**	**3.2 (0.0-331.6)**	**0.020**	**0.71**	**< 1.3**	**91 (21/23)**	**55 (11/20)**

Rv2631	0.0 (0.0-1071)	0.0 (0.0**-**454.8)	0.030	0.69	< 1.3	96 (22/23)	45 (9/20)

**Rv2007c**	**0.0 (0.0-3456.0)**	**0.7(0.0-447.9)**	**0.010**	**0.73**	**< 0.1**	**91 (21/23)**	**55 (11/20)**

Median levels of IFN-γ (pg/ml) and ranges (in parenthesis) elicited in patients with active TB and household contacts by the most promising DosR antigens. Antigens that discriminated between the two groups with P-values < 0.05 are shown. Abilities of the antigens to differentiate between the two study groups are also shown. Antigens in bold discriminated between TB and no TB with AUC ≥ 0.70 after ROC analysis. AUC = Area under the ROC curve

**Figure 2:**
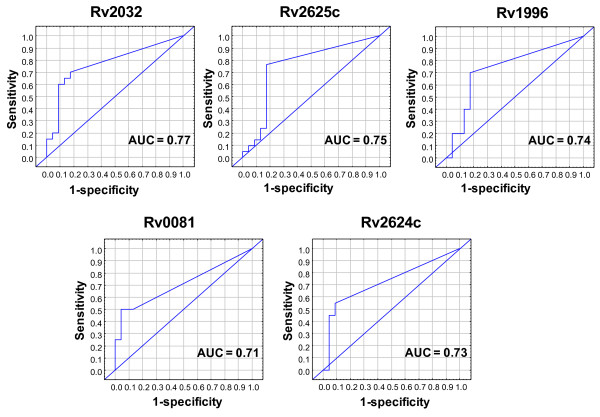
**Receiver operating characteristic (ROC) curves showing the accuracies of the most promising DosR encoded antigens in the diagnosis of TB disease**. Only ROC curves for antigens that differentiated between the TB cases and household contacts with AUCs above 0.71 are shown. ROC curves for all other DosR encoded antigens are shown in Additional file 9: Figure S1. AUC = Area under the curve.

When the data was analyzed by ROC analysis, 8 of the 14 antigens that showed significant differences with the Mann Whitney U test (Rv2625c, Rv1996, Rv2032, Rv0081, Rv2624c,Rv2006, Rv2629 and Rv2007c) differentiated between the two groups with AUCs above 0.70 (Table 1, Figure 2). The diagnostic potentials of all the other DosR encoded antigens evaluated is shown in Additional file 8: Table S8 and the ROC curves are shown in (Additional file 9: Figure S1).

### TB reactivation antigens

A total of 23 TB reactivation associated antigens (Additional file 3: Table S3) were evaluated in this study. All antigens were available as recombinant proteins. Recognition of most of these antigens was poor in both TB patients and HHCs (data not shown). Two of the most frequently recognized of these antigens (Rv1131 and Rv1471) discriminated between the TB cases and HHCs with p values < 0.05. When the data was assessed by ROC analysis, however, only Rv1131 discriminated between the TB cases and HHCs with an AUC ≥ 0.70 (Additional file 8: Table S8, Additional file 9: Figure S1).

### Resuscitation promoting factors (rpfs)

**Figure 3:**
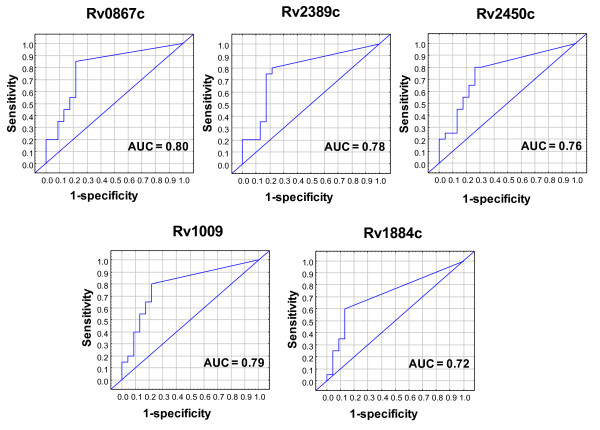
**Receiver operating characteristic curves showing the accuracies of the resuscitation promoting factors in the diagnosis of TB disease**. AUC = Area under the curve.

All of the five known *M.tb *rpfs (Additional file 4: Table S4) were evaluated in this study as recombinant proteins. All rpfs elicited significantly higher IFN-γ responses in HHCs (Table 2) with AUCs ranging from 0.72 to 0.80 (Figure 3) in discriminating TB from non TB.

**Table 2:** Abilities of resuscitation promoting factors (rpfs) to discriminate between TB disease and no TB.

Antigen	TB cases	HHCs	P-value	AUC	Cut-off	Sensitivity (%)	Specificity (%)
Rv0867c	0.0 (0.0-274.6)	52.2 (0.0-2173.3)	< 0.001	0.80	< 1.3	78 (18/23)	85 (17/20)

Rv2389c	0.0 (0.0-209.2)	25.8 (0.0-1056.0)	0.002	0.78	< 0.9	78 (18/23)	80 (16/20)

Rv2450c	0.0 (0.0-209.8)	29.2 (0.0-1123.5)	0.003	0.76	< 10.3	74 (17/23)	80 (16/20)

Rv1009	0.0 (0.0-184.2)	27.2 (0.0-1875.0)	0.001	0.79	< 0.3	78 (13/23)	80 (16/20)

Rv1884c	0.0 (0.0-158.3)	4.0 (0.0-481.1)	0.012	0.72	< 1.3	87 (20/23)	60 (12/20)

Median levels of IFN-γ (pg/ml) and ranges (in parenthesis) elicited upon stimulation of whole blood with rpfs and ability to discriminate between pulmonary TB (in 23 cases) and no TB disease (in 20 HHCs). AUC = Area under the ROC curve

### Starvation antigens and other stress induced proteins

Twenty-four other recombinant antigens, most of which are associated with stress conditions [22] (Additional file 5: Table S5) and 7 starvation induced antigens, all peptide pools (Additional file 6: Table S6), were evaluated in this study. Recognition of these antigens was poor in both TB cases and HHCs. There was no significant difference in the IFN-γ levels elicited by any of the antigens between the TB cases and HHCs (range of p-values, 0.21-0.94). The antigen with the highest AUC for differentiating between the two groups after ROC analysis was Rv0244 (AUC = 0.61). The data indicating the diagnostic potential of these and all the other antigens evaluated in the study is shown in Additional file 8: Table S8 and the ROC curves are shown in (Additional file 9: Figure S1).

### Ability of combinations of antigens to discriminate between TB disease and no TB

**Figure 4:**
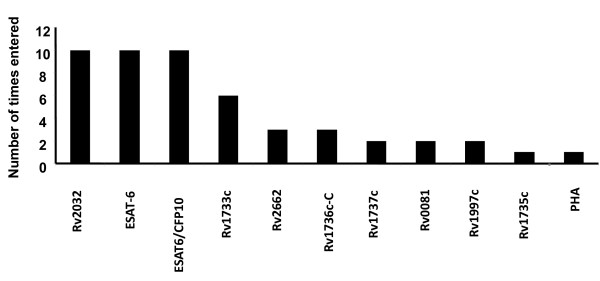
**Number of inclusions of antigens into the 10 general discriminant analysis models that most accurately predicted the presence or absence of TB disease**. PHA was evaluated in all study participants as a positive control, and appeared in one of the models because IFN-γ data was analyzed in a blinded manner.

To investigate whether the discriminative abilities of the antigens could be enhanced when used in combinations, IFN-γ data obtained for all antigens and controls were fitted into general discriminant analysis (GDA) models. Optimal prediction of the presence or absence of active TB was achieved when antigens were used in combinations of four. The most frequently occurring antigens in the top 10 four-antigen combinations that best predicted the presence or absence of TB disease were Rv2032 and ESAT-6/CFP-10fp (or ESAT-6 alone), appearing in all 10 antigen combinations (Figure 4). Addition of two other antigens (Rv1733c + Rv1736c(C-terminal part) or Rv1997c + Rv1733c) to ESAT-6/CFP-10fp and Rv2032 correctly classified 90% of the TB cases and 84% of the HHCs after leave-one-out cross validation.

### Validation of ELISA results with the Quantiferon TB Gold ELISA kit

The standard curve for the ELISA employed in this study ranged from 31 pg/ml to 4000 pg/ml. Although the standard curve was useful at detecting high magnitude IFN-γ responses, IFN-γ responses below 31 pg/ml could not be reliably estimated. As optimal cut-off values obtained for the most promising antigens evaluated in this study (after background correction) were as low as 0.5 pg/ml, we performed validation of the results with another ELISA (the commercially available QFT ELISA kit). Supernatants from the best discriminatory antigens (the top five single DosR antigens (Rv2032c, Rv2625c, Rv1996, Rv0081, Rv2624c) and the top three rpfs (Rv0867c, Rv1009 and Rv2389c)) were re-evaluated. This was done in 15 TB cases and 15 HHCs who were randomly selected from our sample bank. Unstimulated control and ESAT-6/CFP-10fp stimulated supernatants from these 30 subjects were also evaluated.

**Figure 5:**
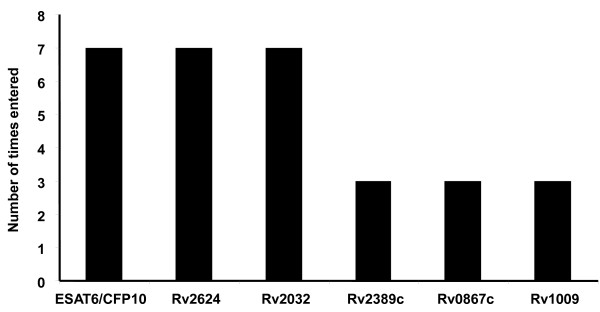
**Number of inclusions of antigens in the 10 most accurate general discriminant analysis combinations that most accurately predicted the presence or absence of TB disease after supernatants were re-evaluated by the Quantiferon TB Gold ELISA**.

Of the nine antigens re-evaluated by the QFT ELISA, significant differences between the TB cases and HHCs were only obtained for the three rpfs (Table 3). Likewise, after ROC analysis, all 3 rpfs discriminated between the TB cases and HHCs with AUCs > 0.73 (Table 3). When the predictive abilities of antigen combinations were evaluated by GDA, the most accurate antigen model (ESAT-6/CFP-10fp + Rv2624c + Rv0867c) accurately classified 83% of all study participants into their respective groups. After leave-one-out cross validation the prediction accuracy was 73% for TB cases and 80% for HHCs (Table 4). The most frequently occurring antigens in the top 10 GDA combinations were ESAT-6/CFP-10fp, Rv2624c and Rv2032 (Figure 5) and all the promising antigen combinations included ESAT-6/CFP-10fp, Rv2624c and one of the three rpfs (Rv0867c, Rv1009, and Rv2389c) (Table 4).

**Table 3:** Abilities of antigens to differentiate between TB disease and no TB disease after re-evaluation with the validation ELISA.

Antigen	TB cases	HHCs	P-value	AUC	Cut-off Value	Sensitivity (%)	Specificity (%)
Rv0867c	1.0 (0-43.8)	25.5 (0-257.5)	0.009	0.78	> 29.3	47 (7/15	93 (14/15)

Rv1009	0.6 (0-27.1)	9.8 (0-273.4)	0.012	0.77	< 1.4	53 (8/15)	87 (13/15)

Rv2389c	0.4 (0-29.5)	23.6 (0-297.4)	0.005	0.80	< 0.1	40 (6/15)	93 (14/15)

Rv0081	0.2 (0-400.0)	1.3 (0-97.1)	0.146	0.65	< 0.1	20 (3/15)	93 (14/15)

Rv1996	0.8 (0-33.7)	0 (0-195.4)	0.771	0.53	> 7.6	20 (3/15)	93 (14/15)

Rv2032	0.5 (0-21.0)	1.3 (0-400.0)	0.120	0.67	< 0.1	47 (7/15)	86 (12/14)

Rv2624c	0 (0-3.7)	0 (0-1.7)	0.295	0.61	< 0.1	27 (4/15)	93 (14/15)

Rv2625c	0.3 (0-31.4)	1.3 (0-400.0)	0.262	0.62	< 0.1	47 (7/15)	87 (13/15)

ESAT-6/CFP-10	305 (0-400.0)	308 (0-400.0)	0.712	0.53	> 441.2	33 (5/15)	96 (26/28)

Median levels of IFN-γ (pg/ml) and ranges (in parentheses) detected in supernatants from TB cases (n = 15) and household contacts (n = 15) after evaluation with the Quantiferon TB Gold ELISA are shown. IFN-γ values were corrected for the unstimulated control levels and responses > 10 IU/ml (400 pg/ml) were given a value of 400 pg/ml. AUC = Area under the ROC curve

**Table 4:** Accuracy of antigens in diagnosing TB disease when used in combinations.

Antigen combination	Resubstitution Classification Matrix			Leave-one-out Cross validation		Wilks lambda	f	P-value
	
	% TB cases	% HHCs	Total %	% TB cases	% HHCs			
ESAT-6/CFP-10 Rv2624c Rv2389c	86.7 (13/15)	73.3 (11/15)	80.0	73.3 (11/15)	73.3 (11/15)	0.59	18.3	0.0002

ESAT-6/CFP-10 Rv2624c Rv0867c	86.7 (13/15)	80.0 (12/15)	83.3	73.3 (11/15)	80.0 (12/15)	0.60	17.5	0.0002

ESAT-6/CFP-10 Rv2624c Rv1009	73.3 (11/15)	73.3 (11/15)	73.3	66.7 (10/15)	66.7 (10/15)	0.62	16.3	0.0004

Rv2032 Rv2624c Rv0867c	80.0 (12/15)	78.6 (11/14)	79.3	80.0 (12/15)	71.4 (10/14)	0.39	38.7	< 0.0001

Rv2032 Rv2624c Rv2389c	86.7 (13/15)	78.6 (11/15)	82.7	86.7 (13/15)	64.3 (9/14)	0.39	39.3	< 0.0001

Rv2032 Rv2624c Rv1009	86.7 (13/15)	71.4 (10/14)	79.3	86.7 (13/15)	64.3 (9/14)	0.41	36.1	< 0.0001

ESAT-6/CFP-10 Rv2032 Rv2389c	80.0 (12/15)	71.4 (10/14)	75.9	80.0 (12/15)	64.3 (9/14)	0.61	15.9	0.0005

ESAT-6/CFP-10 Rv2032 Rv0867c	80.0 (12/15)	78.6 (11/14)	79.3	73.3 (11/15)	71.4 (10/14)	0.62	15.6	0.0005

ESAT-6/CFP-10 Rv2032 Rv1009	73.3 (11/15)	71.4 (10/14)	72.4	73.3 (11/15)	64.3 (9/14)	0.63	14.5	0.0008

ESAT-6/CFP-10 Rv2032 Rv2624c	60.0 (9/15)	64.3 (9/14)	62.1	53.3 (8/15)	64.3 (9/14)	0.67	12.5	0.0015

Abilities of combinations of antigens to discriminate between TB disease and no TB in General Discriminant Analysis models after supernatants were re-evaluated with the Quantiferon TB Gold ELISA. In each case, effect df = 1, error df = 25

## Discussion

The diagnostic potential of 118 *M.tb *infection phase-dependent antigens was investigated in this study. Rpfs were the most discriminatory antigens with combinations of antigens correctly predicting up to 73% of the TB cases and up to 80% of HHCs after cross-validation.

The classical *M.tb *antigens, evaluated here as 'control antigens', have been extensively studied. Most of these antigens are strongly recognized with high IFN-γ production in TB patients or infected individuals [23-28] and are key components of candidate TB vaccines [29-39] or form part of antigen cocktails that are used in diagnostic tests. The frequent recognition of ESAT-6/CFP-10fp (and ESAT-6 alone) in both TB cases and HHCs, justifies the use of these antigens in all commercially available IGRAs. Recognition of TB7.7 (used in the QFT In Tube test, Cellestis, Australia), Ag85A/B and HSP65 was poor. This is in agreement with earlier studies, which suggest that the majority of response to *M.tb *infection is largely driven by ESAT-6 and CFP-10, with TB7.7 providing only a small extra benefit (author's unpublished data). When previously evaluated in serodiagnostic approaches, Ag 85A and B also performed poorly [40] while detection of antibodies or antigens to HSP65 in serum and CSF, respectively, diagnosed active TB with an accuracy above 80% [41,42]. The poor recognition of Ag85A and B, TB7.7 and HSP65 as assessed by IFN-γ production in our study group may suggest that the conditions under which the antigens were tested was not optimal, or perhaps that the humoral and cell mediated responses to these antigens are different. It may also imply that the worth of testing candidate vaccines based on these antigens needs to be reassessed in certain populations. However, it has been shown that responses to TB antigens could be enhanced if antigens are delivered using vectors such as the *Bordetella pertusis *adenylate cyclase vector [43], use of other adjuvants [32] or the addition of IL-7 [44] and IL-12 [45] into cultures. It is not known if addition of cytokines might have improved upon the diagnostic ability of the antigens. Whether some of these antigens would perform better if used in serological assays [41,42] requires further investigation. However, the World Health Organization has recently cautioned against the use of serological tests for the diagnosis of TB disease [46].

The DosR regulon of *M.tb *is upregulated when *M.tb *is subjected to conditions that mimic latency including hypoxia, nutrient starvation, low nitric oxide or low pH [10,19,47-50]. IFN-γ responses elicited by 14 of the DosR antigens evaluated in this study were significantly higher in HHCs compared to TB cases, and 8 of these antigens (Rv2032, Rv2625c, Rv1996, Rv0081, Rv2624c, Rv2006, Rv2629 and Rv2007c) showed promise as diagnostic candidates after ROC analysis, although this promising accuracy was not confirmed by the validation for the five antigens re-evaluated. The inclusion of some DosR regulon encoded antigens into GDA models that best predicted active TB or no TB disease, along with ESAT-6/CFP-10fp or rpfs, may suggest that these antigens are useful diagnostic candidates and need further evaluation in larger studies. The finding that DosR antigens are more frequently recognized in HHCs (many of whom we would expect to be latently infected and all of whom have at least been exposed to *M.tb*) has also been observed in other studies [13,19-21]. Recognition of the DosR antigen Rv2628 was associated with cured tuberculosis and remote infection in a study in Italy [51]. Moreover, an increased frequency of the response to Rv2628 at the site of TB disease in patients with active TB has been found [52].

Like the DosR antigens, the reactivation antigens and the rpfs were more frequently recognized in HHCs, with the rpfs being the most discriminatory between the TB and non-TB cases. Rpfs are growth-promoting proteins and have been shown to resuscitate dormant bacteria, as well as enhance the growth of bacteria, including *M.tb *in both liquid and solid media [53]. *M.tb *rpfs have been shown to be immunogenic in TST-positive individuals and to elicit strong CD4 and CD8 T cell-dependent responses *in vitro*, in long-term latently infected individuals [21,54]. The more frequent recognition of rpfs (believed to be predominantly expressed during the exponential growth phases [53,55]), in latently infected individuals is consistent with the notion that *M.tb *exists in a dormant, non-replicating or only slowly replicating state in LTBI [56,57]. As has been suggested previously, the bacteria may indeed still be replicating but are controlled by the host immune response in an 'active infection' [58]. As suggested by Commandeur et al. [54], the presence of some of the rpfs in culture filtrate may imply higher availability and recognition by immune cells. Furthermore, the existence of *M.tb *in different metabolic states in its host during infection [57] would imply that T cells are exposed (although perhaps in different amounts) to antigens from different infection phases *in vivo*-hence their recognition in *in vitro *stimulation assays. This would also explain the recognition of DosR antigens in TB patients. Whatever the case, antigens that are recognized in both TB patients and latently infected individuals are less well suited for discriminating between different *M.tb *infection states but could add to the currently used RD1 antigens in the diagnosis of *M.tb *infection provided they are potent cytokine inducers in short-term culture assays.

To the best of our knowledge, no other study has investigated such a large number of different *M.tb *infection phase-dependent antigens as diagnostic candidates in a high TB-endemic setting; and little or no information is currently available about the diagnostic potential of most of the antigens investigated in this study. More studies are therefore needed in different population groups to verify the findings of this first screening study. A serious concern in the possible use of new antigens such as those investigated in this study for diagnostic purposes is a lack of specificity for *M.tb*. Some of the DosR antigens evaluated in this study have been shown to be expressed in BCG vaccine strains [47] but BCG vaccination was reported not to induce T cell responses to DosR antigens, likely because BCG fails to establish long-lived latent infections and therefore may not express (or under express) these antigens *in vivo *following vaccination [59].

Although none of the classical TB antigens were capable of discriminating between active TB and no TB, ESAT-6/CFP-10fp was included in most of the top GDA models that best discriminated between the presence and absence of TB disease. It is known that the measurement of IFN-γ elicited by ESAT-6/CFP-10 does not discriminate between active TB and LTBI. However, it has proven to be an excellent marker of *M.tb *infection and this is consistent with the inclusion of ESAT-6/CFP-10fp into all the best discriminatory models. The results suggest that evaluating the DosR antigens or rpfs, which were most predominantly recognized in HHCs, in combination with ESAT-6/CFP-10 could enhance our ability to differentiate LTBI from TB disease in RD1 antigen-positive individuals. When IFN-γ responses against the different antigens were analysed only amongst participants that responded to ESAT-6/CFP-10fp, significant p-values were obtained for all the five rpfs (Rv0867c, Rv2389c, Rv2450c, Rv1009, Rv1884c), 11 DosR encoded antigens (Rv3129, Rv2625c, Rv1996, Rv2032, Rv3126c, Rv2631, Rv3130c, Rv2624c, Rv2007c, Rv3131, Rv3127) and two TB reactivation antigens (Rv1471, Rv3862c). Furthermore, the AUCs for all these 18 antigens in discriminating between TB disease and no TB in the ESAT-6/CFP-10fp responsive participants was above 0.70 (data not shown). Because of the explorative nature of this study, the contributions of the different antigens in the predictive models will need to be investigated further in ongoing follow-up studies.

The main limitations of our study include the relatively small sample size, the use of 7-day WBA instead of overnight assays which would be more suitable for a diagnostic study, and the unavailability of accurate LTBI diagnostic data for the HHCs. The HHCs that participated in this study were recruited from a high TB endemic area [3,60] where it is known that up to 89% of HHCs may be TST positive, with 74% being QFT positive in a previous study [5,13,17]. However the *M.tb *infection status of participants recruited into future follow up studies should be better characterized, so as to enable a more accurate estimation of the discriminative abilities of the antigens between active TB disease and LTBI. This could be done by means of IGRAs and appropriate contact scores based on TB exposure gradient as has been done previously [5,6,61]. For the majority of the antigens evaluated in this study, no information was previously available about their immunogenicity in *M.tb *infected individuals. We therefore chose to evaluate the antigens using a long-term whole blood culture assay in order to detect any existing immune responses to the antigens, including memory responses. Future evaluation of diagnostic candidates should be done in larger numbers of participants, in overnight assays to detect effector cell responses, and preferably using a prospective study design. Potentially useful candidates should also be evaluated in children, immunocompromised individuals, extrapulmonary TB cases and also in individuals with other lung diseases. Because of the need for new TB diagnostic tests to be rapid and less technically demanding, the use of other platforms such as serodiagnosis and the possibility of employing cocktails of antigens should be evaluated. Our data indicates that the two ELISAs employed in this study might not deliver comparable results as only three of the eight promising antigens re-evaluated with the QFT ELISA showed the same promising accuracy obtained with the "BD ELISA". The reasons behind this discrepancy were not investigated further in this study as these technical issues are beyond the scope of this screening study that was designed to identify possible diagnostic antigen candidates. The discrepancies could however, be due to technical aspects that might be inherent in the two ELISAs (the "BD ELISA" with an 8-point 4PL standard curve ranging from 31 pg/ml to 4000 pg/ml versus the QFT ELISA with a 3-point linear standard curve ranging from 0.25 IU/ml to 4.0 IU/ml). ELISA assays used in future studies should be carefully selected, taking into consideration technical aspects including detection limits, to enable more accurate detection of a broad range of IFN-γ responses. Finally, multiplex cytokine analysis assays could be used to evaluate other biomarkers [1,17,62] in supernatants from antigens that did or did not elicit IFN-γ responses since the lack of an IFN-γ response does not necessarily imply that the antigen is not recognized by T cells. The possibility of establishing a useful diagnostic signature comprising a pattern of host responses to different *M.tb *antigens should be investigated.

## Conclusions

In conclusion, IFN-γ responses to TB rpfs associate with latency and show promise as TB diagnostic candidates. The findings of this study are preliminary and require validation in other studies and in other settings.

## Competing interests

The authors declare that they have no competing interests.

## Authors' contributions

KLF and AHF produced all recombinant antigens and TMD produced the peptide pools for the study. THO, TMD, SHEK, MRK, SKP, GW, GFB and NNC helped in designing the study. NNC, GFB, GW, AGL, KS and PNE helped in recruitment of the participants and/or performed the whole blood assays and ELISAs. NNC, GW, THO, GFB, SHEK, TMD helped in analyzing the data or provided intellectual input, and in writing the manuscript. All authors read and approved the final manuscript.

## Pre-publication history

The pre-publication history for this paper can be accessed here:

http://www.biomedcentral.com/1471-2334/12/10/prepub

## Supplementary Material

Additional file 1 **Table S1**. Details of the *M.tb *well-characterized antigens evaluated in the study. *Antigen 85 A and B were tested together in culture, hence assigned the same study code, p = peptide pool. Click here for file

Additional file 2 **Table S2**. Details of the DosR regulon encoded antigens evaluated in the study. Click here for file

Additional file 3 **Table S3**. Description of the TB reactivation antigens evaluated in the study. Click here for file

Additional file 4 **Table S4**. Description of the resuscitation promoting factors evaluated in the study. Click here for file

Additional file 5 **Table S5**. Description of the other *M. tb *stress induced proteins evaluated in the study. Click here for file

Additional file 6 **Table S6**. Description of the starvation antigens evaluated in the study. Click here for file

Additional file 7 **Table S7**. Sequences of the peptide pools evaluated in the study. Click here for file

Additional file 8 **Table S8**. Median levels of IFN-γ (pg/ml) and Inter quartile range (25th to 75th percentile, in parenthesis) elicited upon stimulation of whole blood with different *M.tb *infection phase-dependent antigens and abilities to discriminate between pulmonary TB disease (in 23 cases) and no TB disease (in 19-21 household contacts). AUC = Area under the curve, 95% CI = 95% Confidence interval. The p-values shown are Mann Whitney U test P values for differences between the TB cases and HHCs. The cut-off values are for the sensitivity and specificity for TB disease. Antigens were sorted according to AUC (highest to lowest) for discriminating between TB disease and no TB disease. Click here for file

Additional file 9 **Figure S1**. Receiver operating characteristic curves showing the accuracies of all antigens evaluated in the study in discriminating between TB disease and no TB disease. AUC = Area under the curve. Click here for file
