# Supplementary material for: Potential of novel Mycobacterium tuberculosis infection phase-dependent antigens in the diagnosis of TB disease in a high burden setting
Source: BMC Infect Dis. 2012 Jan 20;12:10. doi: 10.1186/1471-2334-12-10 (PMC3282638; doi:10.1186/1471-2334-12-10)
Supplement: Additional file 9 — Figure S1. Receiver operating characteristic curves showing the accuracies of all antigens evaluated in the study in discriminating between TB disease and no TB disease. AUC = Area under the curve. [file 1471-2334-12-10-S9.DOC]

**Supplementary figure 1**: **Receiver operating characteristic curves for the accuracies of all the antigens evaluated in the study in discriminating between TB disease (in 23 pulmonary TB cases) and no TB disease (in 19 to 21 household contacts).** Area = Area under the curve.
